# Unveiling the magic of mega-city block environments: investigating the intriguing mechanisms shaping children’s spontaneous play preferences

**DOI:** 10.3389/fpsyg.2024.1354236

**Published:** 2024-04-11

**Authors:** Yin Wang, Yinan Sun, Yihao Sun, Ting He

**Affiliations:** ^1^School of Art and Design, Beijing Forestry University, Beijing, China; ^2^School of Architecture, Tsinghua University, Beijing, China

**Keywords:** high-density city, children’s play, child-friendly neighborhood, spatial elements, spatial element preferences

## Abstract

**Introduction:**

This study delves into the spatial preferences of children for play spaces within high-density urban block environments, specifically targeting the area of Baihua Second Road in Shenzhen, China.

**Methods:**

Recognizing the critical role of play in children’s development, and the unique challenges posed by dense urban settings, this research employs multiclass logistic regression models and negative binomial regression models to construct a detailed mathematical analysis of neighborhood spatial elements and children’s play space preferences. Data was meticulously gathered through both objective observations of 14 different types of spaces within the block, and subjective assessments via children’s responses to a series of environment photos, capturing the essence of over 3,000 child participants’ interactions and choices.

**Results:**

Key findings reveal a pronounced preference among children for soft facility features and visually appealing spatial experiences, suggesting a nuanced understanding of play space needs beyond traditional playground designs. Notably, the study identifies that while cartoon-style designs in play facilities might increase moderate attractiveness, ordinary designs hold broader appeal, indicating a preference for diversity in play space aesthetics. These insights offer profound implications for urban planners and designers, advocating for a child-centered approach in the creation of urban play environments that prioritize aesthetic diversity, and the integration of natural elements.

**Conclusion:**

Moreover, the study situates Baihua Second Road as a paradigmatic case, illustrating the methodology and analytical framework applied in addressing the complex interplay between children’s play preferences and urban spatial configurations. By incorporating a comprehensive data-driven analysis, this research contributes significantly to the discourse on child-friendly urban design, offering valuable strategies for cultivating inclusive and engaging urban play spaces for children amidst the constraints of high-density city living.

## 1 Introduction

During a period of rapid social development and transformation, with a global trend of declining birth rates, countries worldwide are increasingly focusing on the healthy growth of children (Schill et al., 2020). Existing research indicates that, apart from the family and school environments, the social microenvironment outside these settings has a crucial impact on children’s development (Twum-Antwi et al., 2020). Therefore, optimizing the environment for children’s growth has become a strategic issue in child development. As the concept of child-friendly cities becomes widely known, the State Council of China issued the “Outline for the Development of Chinese Children (2021–2030)” in September 2021, encouraging the creation of child-friendly cities with public service friendliness, growth space friendliness, and development environment friendliness tailored to China’s conditions (Wang, 2023). In October of the same year, several Chinese government departments, including the National Development and Reform Commission, jointly released the “Guidance on Promoting the Construction of Child-Friendly Cities, “proposing the promotion of child-friendly transformation of public activity spaces such as urban blocks and roads (Nan, 2020). This demonstrates the nation’s high concern for the daily living environment of children and considers meticulous transformation as one of the crucial directions for development. Therefore, focusing on urban blocks and promoting the construction of child-friendly cities is evidently urgent and essential.

Urban block environments are not just traffic channels; they serve as face-to-face communication spaces and social domains for children. They are bridges for creating collective memories and social interactions, the most frequent places for children’s spontaneous play (Guo et al., 2023), and powerful supplements to outdoor play spaces (Min and Lee, 2006). Additionally, they serve as helpful material carriers for the physiological, psychological, social, and educational aspects of children’s growth (Ferrara et al., 2019). Given the complex functional composition of block spaces, the diversity of user types, and their role as secondary activity spaces for children, these environments present unique challenges that must be navigated when children engage in activities within them.

Western countries have long paid attention to the relationship between block environments and children’s play, with research and practical achievements that are rich and systematic (Moore-Cherry, 2014). This has generally experienced three stages: “child health – independence – the relationship between health and the environment (Marzi and Reimers, 2018).” From a spatial planning perspective, this can be summarized as “space demand – independent movement – child participation.” In practice, to provide urban children with public spaces for free play, the UK, supported by local governments, closed blocks to traffic, implementing the radical “play blocks” program (Bertolini, 2020). Although China started later, its development has been rapid, interpreting and adapting theories and examples from Western countries. However, China faces intense contradictions between people and land and features high-rise construction and population density, making the adaptability of successful experiences from Western countries relatively low (Berk, 2015; Dong et al., 2020; Pan et al., 2020). Therefore, Chinese scholars often focus on the characteristics of high-density urban blocks, studying the temporal and spatial characteristics of children’s outdoor activities (Bao et al., 2021), outdoor activity spaces (Gu et al., 2022), construction strategies (Liu et al., 2020), and recreation space planning and construction strategies (Zhang et al., 2022). Existing studies on block environments and children’s play themes focus only on general outdoor activities, neglecting block play behaviors closely related to children’s growth and the environmental spaces that support these activities. Additionally, as a shared space for multiple age groups, the preferences and needs of children in block environments receive little consideration, lacking explanatory power. Even though the child-friendly concept is well-established, attention to children in block environments remains less optimistic. Therefore, this study aims to explore the play behaviors and spatial preferences of children in high-density block environments to understand their true needs. It aims to provide a theoretical basis for the efficient implementation of child-friendly city construction in China, promoting the healthy growth of children.

This study focuses on exploring the formation mechanisms of children’s play space preferences in mega-city block environments, selecting the Baihua Second Road area in Shenzhen City as the specific research field. The concept of a mega-city was first proposed by Hall (1999) as a new urban model emerging in the early 21st century, formed by the extreme diffusion of central large cities to new or neighboring smaller cities (Hall, 1999). Later, the Chinese government and the United Nations Statistics Division further clarified the concept of a mega-city as those large urban areas with extremely high population density and broad geographical range, typically having a permanent population of over ten million. These cities not only exceed the conventional urban boundaries in terms of quantity but also play significant roles in social, economic, and cultural aspects nationally and globally. In terms of research methodology, we adopted a multifaceted methodological framework that combines quantitative and qualitative analyses, aiming to construct a mathematical model of the relationship between block space elements and children’s spontaneous play space choices. Spontaneous play in young children refers to activities conducted during free time where children, driven by their interests and needs, freely choose, initiate, and engage in play for its own sake (Dapp et al., 2021; Sairanen et al., 2022). Initially, by directly observing 14 different types of spaces within the block, we objectively recorded the behaviors of over 3,000 children and collected 143 valid questionnaires on the specific play activities of children. These observations aimed to capture the diversity of spaces and their potential attractiveness to children. Furthermore, to gain deeper insights into children’s subjective preferences for play spaces, we designed a questionnaire survey based on photo selection. In this survey, children were invited to choose their preferred play spaces based on their reactions to environmental photos. These photos included both those collected from our field observations and those obtained from the internet related to the research area, ensuring the research data’s breadth and diversity. By combining these two research methods—objective observation of spaces and children’s subjective choices—this study aims to comprehensively capture and analyze the various factors influencing children’s play space preferences. Data analysis primarily relied on multinomial logistic regression models and negative binomial regression models to reveal the intrinsic connections between block space elements and children’s play space choices. Additionally, special attention was given to the scientific integrity and reliability of the data, ensuring the research hypotheses were supported through rigorous statistical analysis methods.

The main contributions of this study are as follows: (1) Through in-depth research on the empirical case of Baihua Second Road in Shenzhen, mathematical analysis models were constructed for the spatial elements of block environments and children’s choices in play spaces using multiclass Logistic regression models and negative binomial regression models. This model is not only methodologically innovative but also provides a comprehensive and profound understanding of children’s play behavior in high-density urban block environments. (2) This study, within the framework of environmental psychology, delves into the preferences of children for spatial elements in high-density block environments. Through the analysis of children’s consistent and personalized preferences for different spatial elements, we provide robust support for understanding the psychological mechanisms of children’s interaction with the environment. (3) The study not only presents empirical results on children’s play behavior and spatial preferences but also proposes strategic recommendations, including the top priority of avoiding blindly pursuing excessive childlike elements, and encouraging the coexistence of informal and formal play spaces. These suggestions fundamentally integrate the principles of environmental psychology into the practice of urban planning and design, providing operational guidance for creating urban spaces that better meet the needs of children. (4) The study provides a new perspective for the research on block environments and children’s play in high-density Chinese cities, injecting new empirical data and practical knowledge into the field of environmental psychology. It promotes the continuous evolution of child-friendly city construction in China and offers inspiration for future global research.

The remainder of this study is organized as follows. Section 2 analyzes children’s play and their preferences for urban block environmental features. Section 3 introduces the research design, including data sources and the experimental design process. Section 4 details the construction of models and data analysis regarding children’s play choices and spatial elements. Section 5 discusses in detail the relationship between children’s play choices in block spaces and spatial elements. Section 6 proposes construction strategies for child-friendly neighborhoods based on the results of children’s play space preferences. Section 7 presents the study’s conclusions and limitations.

## 2 Children’s play and preferences for block environmental elements

### 2.1 Children’s play in mega-city block environments

The process of high-density development in mega-cities typically involves increasing population and residential density within urban areas (Jenks et al., 1996). Residents face conflicts between limited physical space and a dense population (Hall, 2014). Children, more sensitive than other groups, experience the negative impacts of high-density urban development (Bowler et al., 2010). Independent play spaces for children in mega-cities are extremely limited, despite designers creating various play spaces. Surprisingly, studies show that children do not consider playgrounds their favorite play areas (Egli et al., 2020). Instead, ordinary urban environments are crucial play spaces for children in high-density cities. A survey by Man (1996) found that children spend more time playing on blocks than in parks, with blocks and intersections being important meeting places for them. Urban blocks are not just traffic channels; they are vital spaces for children’s communication, socialization, and spontaneous play (Özbayraktar, 2016). Community blocks become essential spaces for children’s play, socialization, and growth in high-density cities. However, rapid industrial and technological development has led to a rapid increase in vehicles, squeezing the block spaces where children can freely play (Miller, 1969). Traffic-related risks remain a major concern for parents.

Moreover, adults have built parks, playgrounds, and other game spaces to prevent children from playing in inappropriate places (Rasmussen, 2004). However, scholars argue that creating specific spaces for children may isolate them from other urban environments (Goodman, 1979). Researchers found that children perceive elements in block environments as props for games during their commute, promoting cognitive and social development (Manikam and Said, 2016; Chatterjee, 2018). As a complex space shared by multiple age groups and various modes of transportation, the block has become an indispensable part of children’s daily lives, particularly charming yet hazardous for their play activities. We cannot deny the crucial role of block spaces in the play of children in mega-cities. Therefore, understanding the correlation between children’s play and block environments is essential for guiding children’s healthy play behavior.

### 2.2 Children’s play and spatial preferences

Spatial preferences refer to the value judgments individuals or groups make about spatial attractions and choices. It involves the value judgments individuals or groups make about entering or selecting a space based on the attraction of things within that space. Conversely, through the value judgments made by entering or selecting a space, their perceived preferences for that space are analyzed (Haans, 2014; Cho and Suh, 2020).

From the perspective of urban design, scholars have studied the construction of children’s behavior and spatial preferences, confirming a correlation between children’s behavior and block material elements. The type and characteristics of space can influence children’s spatial preferences. For example, beautiful environments, rich play facilities, and good maintenance are attractive to children, followed by convenient transportation and more peers (Marcus and Sarkissian, 2023). Ekawati found that traffic stability has the most significant impact on children’s outdoor activities in blocks, while the impact of facilities and materials is minimal, followed by scale and layout, green spaces, play areas, and accessibility (Ekawati, 2015). Liu Kun and Wei Zijun pointed out that factors such as commercial activities along blocks, interface permeability, and rich social activities on blocks provide important interaction interfaces for children’s block life, promoting their block activities (Liu and Wei, 2019).

Additionally, children have unique cognition and preferences during play activities, often differing from parents and designers. Research indicates that children’s vision differs from that of adults. From the age of 3, visual neural development gradually matures, making children highly sensitive to visual perceptions, sparking increasing curiosity and a desire for exploration (Mangal and Mangal, 2019). This curiosity and desire for exploration can stimulate spontaneous play behavior in children. Therefore, children’s play preferences are significantly influenced by visual perceptions (Egli et al., 2020). Furthermore, Gibson, from the perspective of environmental behavior, believes that the frequency of children using a place can be used to infer whether they like that place (Barker and Wright, 1955).

### 2.3 Environmental elements

Environmental elements are the basic components of environmental space. Whether formal children’s play areas or informal play spaces in urban blocks, they can be broken down into basic environmental elements (del Pulgar et al., 2020). Children do not distinguish between the nature of spaces when choosing play spaces in blocks. This study, considering the comprehensiveness of children’s preferences, does not distinguish between formal and informal spaces. Instead, it deconstructs the block environment as a whole and uses environmental elements as evaluation indicators for children’s visual perceptions.

Existing research in this field mainly focuses on outdoor activities and interaction behaviors of children, while studies on the correlation between children’s play and related elements are relatively lacking. According to Ding and Han (2019) and Lin and Guo (2022), the environmental elements affecting children’s play preferences are the basic elements constituting block play spaces. Therefore, this study classifies the basic elements of block environments into built environment elements and other social elements, totaling 9 environmental elements and 20 influencing factors. To better analyze space characteristics, this study summarizes and classifies the elements that make up space into seven primary indicators, including space type, space color, space texture, building type, naturalness of space, facility details, and space users. It also includes secondary indicators such as facility type, facility shape, facility color, ground paving, space size, plants, animals, peers, parents, etc. (Table 1). Through children’s subjective spatial preferences and objective play behavior, this study explores the correlation between children’s play and environmental elements. It aims to understand their perceptions and preferences for block play spaces, analyze the environmental elements influencing their choices, and effectively comprehend children to construct block environments that better meet their needs.

**Table 1:** Classification of indicators of spatial components.

Level 1 indicators	Level 2 indicators	Type of indicator
Space Type X1	Space type x1	Sequential Data
	Space size x2	Sequential Data
Space Color X2	Environment color x3	Sequential Data
	Facility vignette color x4	Sequential Data
Space Texture X3	Pavement x5	Sequential Data
	Facility Vignette Texture x6	Sequential Data
Facility vignette X4	Playfulness x7	Sequential Data
	Facility Vignette Styling x8	Sequential Data
Building type X5	Building type x9	Sequential Data
Naturalness X6	Vegetation x10	Sequential Data
	Terrain x11	Sequential Data
User X7	Children x12	Sequential Data
	Adults x 13	Sequential Data

## 3 Research design

**Figure 1:**
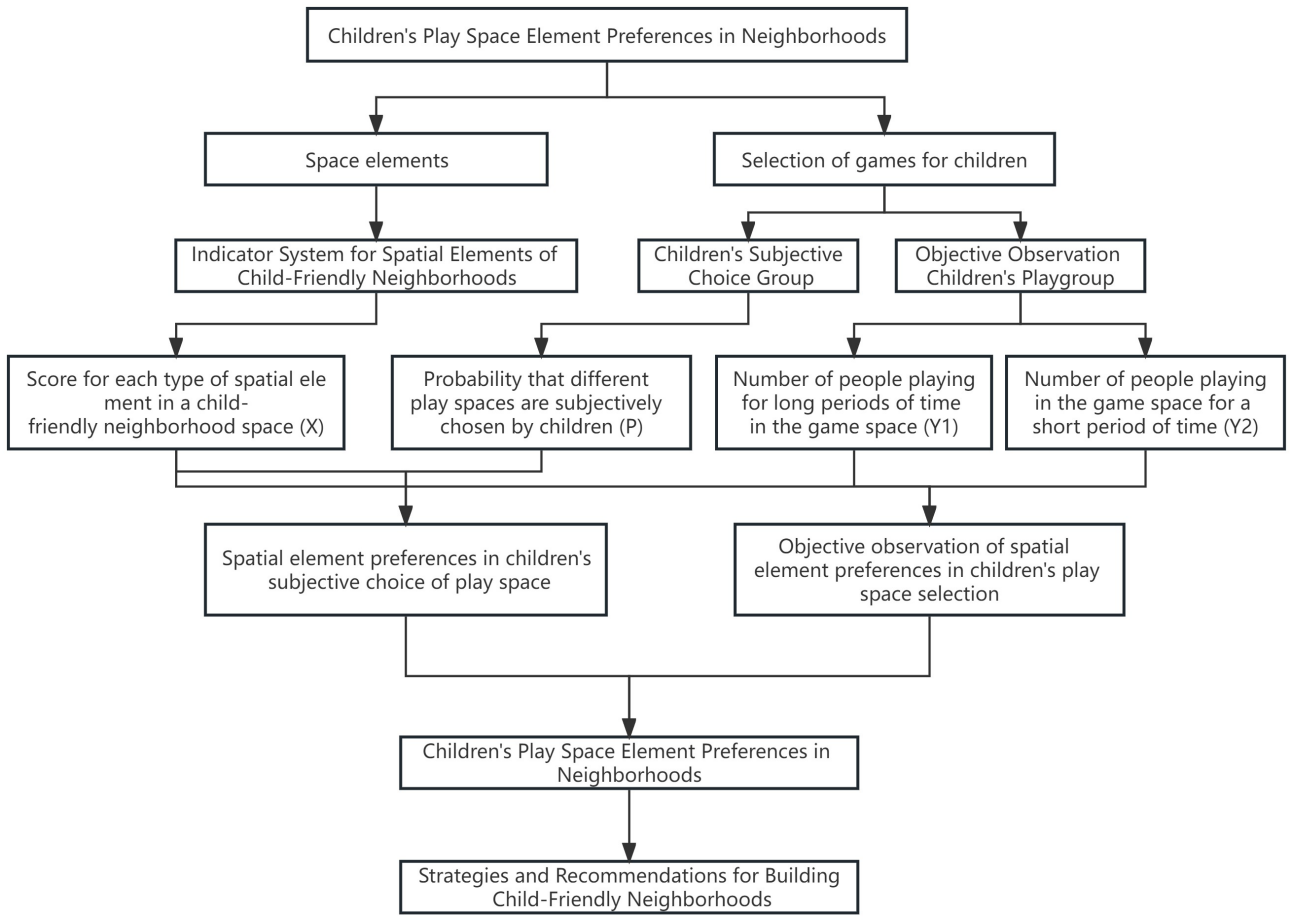
Research framework.

This study establishes the relationship between spatial elements and children’s choices of play spaces. By analyzing models, it aims to obtain the mathematical relationship between the two and understand children’s spatial element preferences when selecting play spaces. Current studies of a similar nature typically rely on either single-objective observations or subjective data from children’s choices (Barker and Wright, 1955; Mangal and Mangal, 2019; Egli et al., 2020). However, as children’s outdoor activities are to some extent influenced by parental guidance, objectively observed data may not entirely reflect children’s autonomous choices. On the other hand, while children’s subjective choices through viewing photos or videos ensure independent decision-making, there is an issue of children having a vague understanding of the chosen materials. To comprehensively understand children’s spatial preferences for play in child-friendly neighborhoods, this study constructs a research framework that combines children’s subjective choices with objective PSPL (Photo Sort and Paired Comparison) survey data. Models are built for the collected data, and the results are compared and analyzed to comprehensively and objectively reflect children’s spatial preferences for play (Figure 1).

### 3.1 Research environment

**Figure 2:**
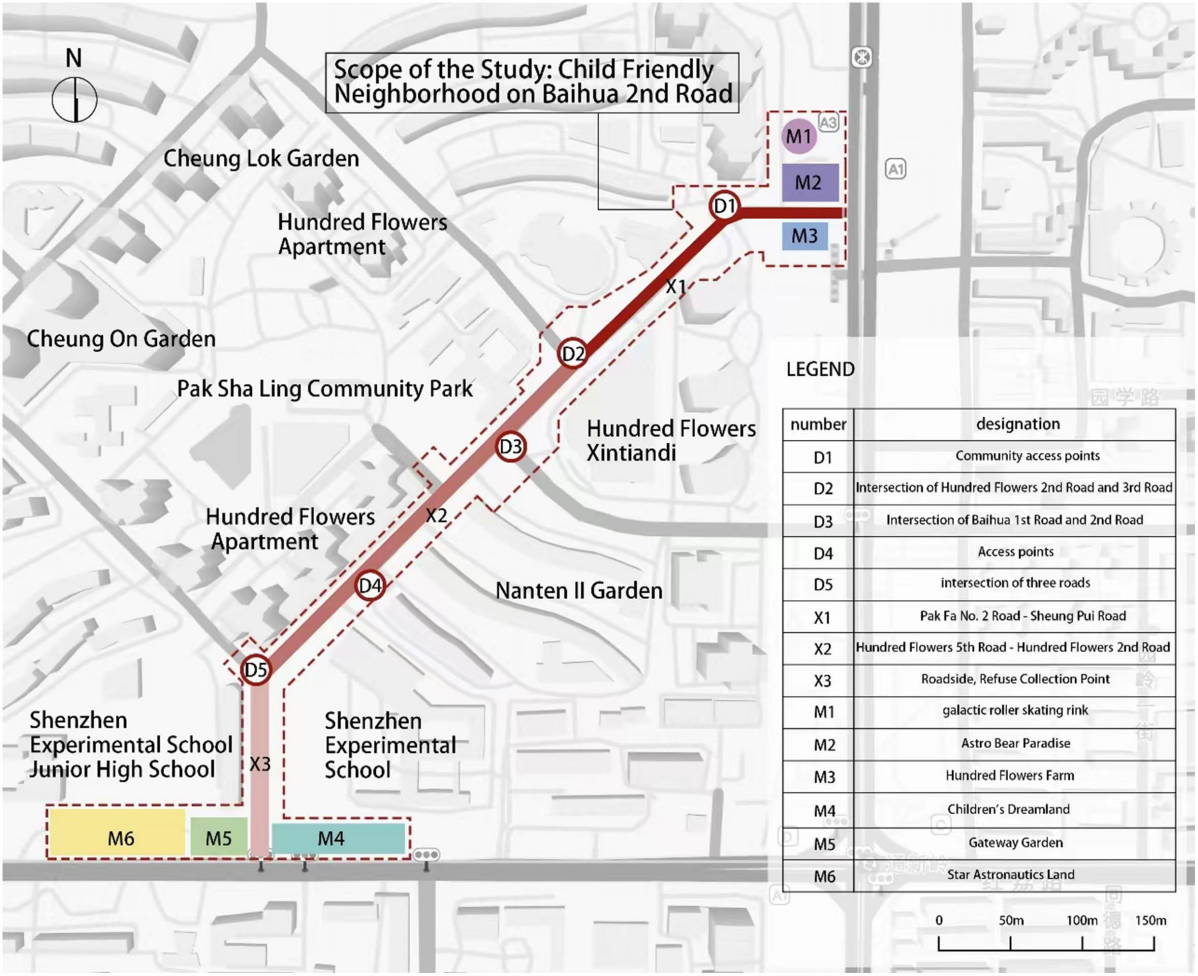
Research scope.

For this study, Shenzhen’s Baihua Er Road Child-Friendly Block was chosen as the research objectives. Shenzhen, as a high-density city, early on proposed and implemented plans for building a “child-friendly city” and demonstrated the construction of a child-friendly city with Chinese characteristics (Bhuyan and Zhang, 2020). Baihua Er Road is one of Shenzhen’s first child-friendly blocks, approximately 750 meters long, surrounded by 11 prestigious primary and secondary schools, serving as a benchmark education base in Shenzhen. The area accommodates around 3,500 children aged 4–6 and over 10,000 youths aged 7–14. Besides the regular block traffic space, the child-friendly block provides formal play areas for children, including Gateway Gardens, Mind Corners, Dreamland, Baihua Children’s Music Theater, Variations Wall, Roller Skating Arena, and Baihua Farm (Figure 2). The entire block offers diverse spatial types, making it highly suitable as the research objectives.

### 3.2 Experimental design

This study employs an empirical research method that divides data into subjective and objective groups, aiming to fully capture and understand the multidimensional characteristics of the research subject. The objective data group provides reliable, quantified characteristics of behavior and environment through direct observation and recording, ensuring the empirical nature and accuracy of the research findings. Meanwhile, the subjective data group collects participants’ personal feelings and preferences through surveys and other means, reflecting the internal psychological and emotional dimensions of the research subject (Cabedo-Mas et al., 2021; Alexander et al., 2024). This method of combining subjective and objective data not only allows for a comprehensive understanding of the research topic from both external behaviors and internal perceptions but also helps to reveal the motives and preferences behind behaviors, offering richer and more in-depth insights for the study. Additionally, because the objective observation of children’s play behavior might be influenced by parental involvement rather than the children’s own choices (Šimunović and Babarović, 2020; Wendel et al., 2020). Subjective questions, due to children observing pictures, may lead to certain misjudgments. Moreover, the application of this methodology also increases the complexity and challenge of the research but significantly enhances the validity and applicability of the results, contributing to a more comprehensive and integrated research conclusion.

In the work of the objective research group, we selected 14 representative spaces within the block based on conditions such as “comprehensive space types and distinctive features.” These spaces were subdivided into point spaces (5 locations), strip spaces (3 locations), and area spaces (6 locations), according to their functional and morphological characteristics. Specifically, “point spaces” refer to small areas with specific functional points, such as individual facilities within playgrounds; “strip spaces” are elongated areas like sidewalks that connect different point spaces; and “area spaces” refer to larger open areas, such as parks or squares. This distribution aims to comprehensively cover different types of activity areas within the block, ensuring the research’s breadth and depth. To improve efficiency and accuracy in data collection and analysis, we limited the spatial elements of the objective group to primary indicators and used expert scoring to rate each research space’s relevant characteristics. The scoring criteria included: based on actual observations, “space color,” “space texture,” and “naturalness” were each assigned a score of 1 to 3 to reflect the environmental characteristics of each space; the presence of “facility vignettes” was simplified to a yes (1 point) or no (0 points) judgment; meanwhile, “space type” and “building type” were classified according to their actual characteristics.

Since it’s challenging to distinguish whether the “users’ activities” were children playing, we did not consider whether the users were children engaged in play as a direct factor and mainly relied on non-intrusive observations to gather data. To ensure the data were as comprehensive and accurate as possible, we established 5 observation points with good visibility and continuity around the 14 activity spaces. Regular recordings of at least 10 min were made at different observation points every hour, detailing the number of children playing, types of behavior, and duration. Additionally, to comprehensively reflect children’s play behavior during different seasons and times, considering Shenzhen’s humid and hot geographic environment and high-density residential characteristics, observations were arranged in both summer and winter, weekdays and weekends, from 9 a.m. to 10 p.m. every day, distinguishing between brief stays and long-duration play behavior with a 5-min criterion. This classification helps us more accurately understand children’s play preferences and behavior patterns in block spaces.

In the subjective study part, we distributed 50 environmental photos of the 14 spaces selected by the objective group and 30 relevant activity scene photos collected from the internet to children. The children were invited to select their preferred play space photos through unlimited voting, and then the vote counts for each photo space were tallied. To ensure the selected photos fully reflected the characteristics of each site, research team members made targeted choices during the preliminary selection stage and performed detailed identification and rating of the environmental elements in the photos according to secondary indicators. This included assigning scores of 1 to 3 for ordinal data and specific classification for categorical data.

The study focused on children aged 6 to 14, recording behaviors of over 3,000 children through objective observation and successfully collecting 143 valid questionnaires in the subjective assessment part. The design and execution of this methodology aim to ensure the comprehensiveness, accuracy, and reliability of the research results, providing a solid data foundation for subsequent analysis.

## 4 Model construction and data analysis of children’s game selection and spatial elements

### 4.1 Objective group

Through observations of the objective group, it was identified that children’s primary game activities in the 14 spaces can be categorized into short-duration games and long-duration games. Short-duration games involve brief stops, window shopping, skateboarding, running, and other fast-paced activities, while long-duration games include sports, conversations, and playing with play activities facilities. The number of participants in short-duration games (Y2) is fewer than the number in long-duration games (Y1).

A model with six primary indicators (X) was constructed to analyze the data related to the number of participants in long-duration games (Y1) and short-duration games (Y2). Dummy variables were introduced for categorical data to enhance the interpretability of the regression. Since Y1 and Y2 are count data types, after model construction, the absolute value of the odds ratio (OR) was found to be greater than 1.96, indicating that a negative binomial regression model is suitable for comparison.

Using SPSS to build the mathematical model, it was found that the *p*-value for the model of Y2 with the primary indicators of spatial elements was greater than 0.05, indicating no significance. This suggests that spatial elements do not have a significant impact on Y2, indicating that children do not have specific spatial element preferences for short-duration game activities.

For Y1, the model with X had a *p*-value of 0.00, indicating model effectiveness (see Table 2). The model formula is as follows Equation (1):

(1) $$\begin{array}{l} \log\left(Y\right)=-3.395-0.710\times 2+2.692^{*}X3+0.235X1a-\\ 2.293X1b-1.337X1c+1.126X5a-\\ 2.430X5b-1.326X5c+1.354^{*}X6+1.009\times 4 \end{array}$$

**Table 2:** Summary of negative binomial regression model results (*n* = 42).

Term	Regression coefficient	Standard error	*z*-value	*p*-value	OR-value	OR 95% CI
Intercept	−3.395	1.615	−2.102	0.036	0.034	0.001 ~ 0.795
Point space X1a	0.235	0.829	0.283	0.777	1.265	0.249 ~ 6.423
Line space X1b	−2.293	0.949	−2.416	0.016	0.101	0.016 ~ 0.648
Surface space X1c	−1.337	0.771	−1.734	0.083	0.263	0.058 ~ 1.190
Space color X2	−0.710	0.384	−1.851	0.064	0.492	0.232 ~ 1.043
Space Texture X3	2.692	0.710	3.792	0.000	14.762	3.671 ~ 59.357
Facility Vignette X4	1.009	0.598	1.688	0.091	2.743	0.850 ~ 8.850
Commercial Building X5a	1.126	1.031	1.093	0.275	3.084	0.409 ~ 23.251
Education X5bfere	−2.430	0.685	−3.548	0.000	0.088	0.023 ~ 0.337
Residential X5c	−1.326	0.538	−2.464	0.014	0.265	0.092 ~ 0.763
Naturalness X6	1.354	0.357	3.796	0.000	3.872	1.925 ~ 7.789

The study reveals that the *p*-values for Tactile Sensation (X3), Linear Space (X1b), Educational Buildings (X5b), Residential Buildings (X5c), and Natural Attributes (X6) are less than 0.05, indicating a significant impact on the results for the number of participants in long-duration games. The regression coefficients for Spatial Sensation and Natural Attributes are positive, suggesting that soft spatial sensations are more attractive to children for extended gameplay compared to rigid spatial sensations, and a higher degree of naturalness corresponds to more participants in long-duration games. The regression coefficients for Linear Space, Educational Buildings, and Residential Buildings are negative, implying that linear spaces and spaces surrounded by educational or residential attributes are relatively unattractive for children to engage in long-duration play.

### 4.2 Subjective group

The number of selections for each photo in the subjective group was transformed into the probability of a space being chosen (*R*). It was observed that the selection probabilities primarily concentrated between 0 and 85%. Therefore, selection probabilities were defined as low attractiveness for 0–25%, moderate attractiveness for 25–50%, and high attractiveness for over 50%. A multinomial logistic regression model was constructed to analyze the relationship between the probability of space selection (R) and the secondary indicators (x) of the 14 spatial elements (see Table 3). Introducing dummy variables for categorical data, the model’s p-value was 0.000, less than 0.05, indicating statistical significance. The model formula is denoted as follows Equations (2, 3):

(2) $$\begin{array}{l} \ln\left(2.0/1.0\right)=0.425-0.367x_{7}-2.106x_{8}+0.410x_{6}+\\ 0.499x_{4}+0.699x_{1a}-0.257_{x1b}+\\ 1.259_{x1c}+0.382x_{2}+1.078x_{3}+\\ 0.817x_{5}-1.140x_{9a}-2.109x_{9b}-\\ 1.214x_{9c}-1.343x_{10}+0.327x_{11}+\\ 5.688x_{12}-3.027x_{13} \end{array}$$

(3) $$\begin{array}{l} \ln\left(3.0/1.0\right)=4.144-1.495\;x_{7}+5.805\;x_{8}+1.879\;x_{6}-\\ 1.553\;x_{4}+8.531\;x_{1a}+5.533_{x1b}\\ +2.512_{x1c-3}.387\;x_{2}+1.440\;x_{3}\\ -1.204\;x_{5}+1.330\;x_{9a}+0.383\;x9b-\\ 27.773\;x_{9c}-0.267\;x_{10}-4.736\;x_{11}+\\ 3.525\;x_{12}-0.894\;x_{13} \end{array}$$

**Table 3:** Summary of multinomial logistic regression model results.

2.0	Regression coefficient	Standard error	*z*-value	Wald *χ*^2^	*p*-value	OR-value	OR 95% CIfere
Point space x1a	0.699	8575933.998	0.000	0.000	1.000	2.012	0.000 ~ Infinity
Line space x1b	−0.257	8580701.493	−0.000	0.000	1.000	0.773	0.000 ~ Infinity
Surface space x1c	1.259	8572640.692	0.000	0.000	1.000	3.521	0.000 ~ Infinity
Space size x2	0.382	1.610	0.238	0.056	0.812	1.466	0.062 ~ 34.409
Environment color x3	1.078	0.880	1.226	1.502	0.220	2.940	0.524 ~ 16.494
Facility vignette color x4	0.499	0.741	0.673	0.453	0.501	1.647	0.385 ~ 7.040
Pavement x5	0.817	0.818	0.998	0.996	0.318	2.263	0.455 ~ 11.247
Facility vignette texture x6	0.410	0.696	0.589	0.347	0.556	1.507	0.385 ~ 5.897
Playfulness x7	−0.367	0.873	−0.420	0.176	0.675	0.693	0.125 ~ 3.838
Facility vignette styling x8	−2.106	1.009	−2.086	4.352	0.037	0.122	0.017 ~ 0.880
Commercial buildings x9a	−1.140	1.882	−0.606	0.367	0.545	0.320	0.008 ~ 12.794
Residential buildings x9b	−2.109	1.254	−1.682	2.827	0.093	0.121	0.010 ~ 1.418
Educational buildings x9c	−1.214	1.418	−0.856	0.733	0.392	0.297	0.018 ~ 4.783
Vegetation richness x10	−1.343	0.759	−1.768	3.128	0.077	0.261	0.059 ~ 1.157
Terrain complexity x11	0.327	1.047	0.312	0.097	0.755	1.387	0.178 ~ 10.803
Children x12	5.688	2.357	2.413	5.823	0.016	295.209	2.910 ~ 29950.372
Adults x13	−3.027	1.794	−1.687	2.846	0.092	0.048	0.001 ~ 1.632
Intercept distance	0.425	34307617.953	0.000	0.000	1.000	1.530	0.000 ~ Infinity
3.0	Regression Coefficient	Standard Error	z-value	Wald χ2	p-value	OR-value	OR 95% CI
Point space x1a	8.531	8487429.000	0.000	0.000	1.000	5069.830	0.000 ~ Infinity
Line space x1b	5.533	8487429.000	0.000	0.000	1.000	252.950	0.000 ~ Infinity
Surface space x1c	2.512	8487429.000	0.000	0.000	1.000	12.334	0.000 ~ Infinity
Space size x2	−3.387	2.088	−1.623	2.633	0.105	0.034	0.001 ~ 2.022
Environment color x3	1.440	0.942	1.528	2.335	0.127	4.221	0.666 ~ 26.767
Facility vignette color x4	−1.553	1.156	−1.343	1.803	0.179	0.212	0.022 ~ 2.041
Pavement x5	−1.204	1.404	−0.858	0.735	0.391	0.300	0.019 ~ 4.700
Facility vignette texture x6	1.879	0.936	2.008	4.033	0.045	6.550	1.046 ~ 40.997
Playfulness x7	−1.495	1.074	−1.392	1.939	0.164	0.224	0.027 ~ 1.839
Facility vignette styling x8	5.805	2.977	1.950	3.803	0.051	331.979	0.971 ~ 113473.077
Commercial buildings x9a	1.330	1.892	0.703	0.494	0.482	3.780	0.093 ~ 154.026
Residential buildings x9b	0.383	1.617	0.237	0.056	0.813	1.467	0.062 ~ 34.900
Educational buildings x9c	−27.773	18210.709	−0.002	0.000	0.999	0.000	0.000 ~ Infinity
Vegetation richness x10	−0.267	1.036	−0.258	0.066	0.797	0.766	0.101 ~ 5.828
Terrain complexity x11	−4.736	2.673	−1.772	3.140	0.076	0.009	0.000 ~ 1.653
Children x12	3.525	2.458	1.434	2.057	0.152	33.956	0.275 ~ 4198.300
Adults x13	−0.894	2.002	−0.447	0.200	0.655	0.409	0.008 ~ 20.673
Intercept distance	4.144	33949707.929	0.000	0.000	1.000	63.065	0.000 ~ Infinity

Using low attractiveness (1.0) as a reference, when aiming to elevate the attractiveness of a space to moderate (2.0), “Facility Ornamentation” and the presence of “Children” have a significant impact. The regression coefficient for “Facility Ornamentation” is negative, indicating that the more cartoon elements the ornamentation possesses, the higher the attractiveness. The regression coefficient for “Children” is positive, suggesting that the presence of children playing in the area enhances its attractiveness.

However, if low attractiveness (1.0) is taken as a reference, and the goal is to raise the attractiveness to high (3.0), “Facility Ornamentation” and “Facility Texture” have a significant impact. The influence of “Children” on attractiveness does not have a significant impact. The positive regression coefficient for “Facility Ornamentation” implies that if aiming for high attractiveness, cartoon-style facility ornamentation may decrease the overall attractiveness of the space, while standard designs significantly positively influence children’s choices. The positive regression coefficient for “Facility Texture” indicates that when striving for high attractiveness, facilities with a soft texture are more appealing to children.

## 5 Discussion on the research results of children’s game space selection and spatial elements in block spaces

Integrating data analyses from both subjective and objective groups reveals significant differences in influential environmental factors. This underscores the limitations of a singular research method and highlights the importance of combining subjective and objective analyses.

### 5.1 Common preferences in long-term game selection in block spaces

Both subjective and objective analyses emphasize the significant impact of several spatial elements on children’s game space selection. This indicates the crucial role of spatial elements in shaping children’s game space preferences and influencing their play activities behavior to some extent. Children exhibit distinct preferences for specific spatial elements, providing valuable guidance for future urban planning and child-friendly environmental design. Firstly, in game space selection, children show a preference for soft facilities and visual spatial experiences (Jiang, 2020). Subjectively, under high attractiveness conditions, children tend to choose facilities that appear soft rather than rigid, offering robust recommendations for designing game spaces with soft elements. Objective observations also confirm a positive correlation between soft spaces and an increase in the number of long-term players, further validating children’s actual inclination toward soft spatial elements. Secondly, the study finds that children have preferences for facility design. Facilities with cartoon-style designs are more likely to become moderately attractive spaces, while those with ordinary designs are more widely preferred. This discovery, differing from the common perception of strong attraction to cartoon-style designs (Gomes, 2021), suggests a need to focus on integrating and balancing various design elements when creating children’s game spaces, fostering a more appealing play activities environment.

### 5.2 Influence of parents and peers on long-term game space selection in block spaces

The disparities between subjective and objective results reveal significant differences in children’s game space selection based on subjective choices and the influence of parents. Parents are influenced by various factors when guiding their children’s choices, including the type of space, surrounding building types, and the richness of the natural environment (Loebach and Gilliland, 2022). Parents are reluctant to let children engage in activities in linear spaces with heavy pedestrian traffic and avoid selecting areas near educational and residential buildings, possibly due to these places serving as destinations for students commuting to and from school. In contrast, parents tend to choose places with a better natural environment, possibly because they consider the natural surroundings to be essential for children’s development (Chawla, 2020), and such environments provide parents with relatively comfortable supervision. However, these factors do not exhibit a significant impact on children’s subjective choices. Additionally, from children’s subjective choices, the presence of playing peers tends to increase the probability of a place being chosen, while the presence of parents or adults does not significantly affect the selection probability. This suggests that, for children, the presence of peers playing in the area has a certain attraction, while the presence of adults does not significantly influence children’s choices. However, it is important to note that if the attractiveness of the location is already high, the presence of peers playing may no longer have a significant impact, indicating that not all children prefer lively game spaces with companions. This finding contributes to a more comprehensive understanding of children’s game space selection in different contexts, providing valuable insights for future child-friendly space design.

### 5.3 Strong randomness and individual preferences in children’s game space preferences in block spaces

While we have identified some environmental elements that impact children’s game space selection, it is noteworthy that most spatial elements do not have a significant effect on both subjective and objective choices for long-term and short-term games. This suggests that children’s choices during play reflect more individualistic characteristics, demonstrating strong subjective preferences. This phenomenon succinctly reveals children’s curiosity and willingness to explore different choices during their developmental stage without consciously filtering environmental elements. In contrast, adults, after forming aesthetic habits and considering risk and benefit factors, develop relatively fixed behavioral experiences influenced by societal norms (Maiese, 2022), particularly evident in their aesthetic preferences for spatial elements (Hubbard, 2018). However, children’s choices tend to rely on genuine inner feelings, not yet forming a fixed aesthetic orientation, presenting diverse characteristics. This challenges traditional perceptions of “childish” demands in designing children’s play areas, such as the need for colorful, cartoonish, or facility-centric elements. Instead, it suggests a deeper understanding that children’s choices for block game spaces exhibit strong randomness and individuality. This recognition poses new challenges for future urban planning and child-friendly environmental design, requiring a more flexible approach to cater to the personalized and diversified needs of children during play.

## 6 Construction strategies for child-friendly blocks based on children’s game space preferences

From the experimental results, it is evident that children’s game space choices in the block are to some extent influenced by spatial elements, providing reference for improving block environments for urban developers. By optimizing the spatial environment of block spaces to some extent, the block can become more attractive, and more vibrant. Therefore, based on the research results, this paper proposes three constructive strategies for constructing child-friendly blocks in large cities.

### 6.1 Enhancing exploration and engagement in child-friendly block spaces

In mega-cities, the construction of child-friendly neighborhoods should focus on fostering environments that stimulate exploration and engagement over mere aesthetic appeal. Our research highlights that children’s interaction with play spaces often transcends the structured designs envisioned by planners, revealing a penchant for spaces that ignite their curiosity and encourage spontaneous play. This observation underscores the importance of designing urban environments that cater to the intrinsic exploratory and playful instincts of children rather than confining them to predetermined play areas. To achieve this, urban designers and managers are encouraged to adopt flexible design philosophies and management strategies that prioritize the creation of versatile and engaging play environments. Such environments should offer a variety of play elements and themes to cater to the diverse interests and developmental needs of children across different age groups. Moreover, the design process should consider the integration of features that allow for both guided and autonomous play, thereby fostering a sense of discovery and personal growth among young city dwellers. Innovative design concepts might include the use of natural landscapes and elements to create multifunctional play areas that encourage children to interact with their surroundings in meaningful ways. Additionally, incorporating adaptable play structures that can be used in multiple ways can further enhance children’s creative engagement and social interaction within these spaces.

### 6.2 Avoid blindly pursuing “child-friendly” elements in block spaces for children’s games

In discussing the design of children’s play spaces, we discovered that children’s preferences for spatial elements show diversity rather than a singular trend towards “childization” and “gamification.” This suggests that in design, we should avoid the application of uniform and overly “childized” or “gamified” elements, and instead adopt a more diverse and inclusive design strategy. The design should focus on creating various types of play spaces that cater to children’s preferences for soft materials and encourage them to engage in a variety of play behaviors, promoting their personalized development. Furthermore, considering the influence of families on children’s play environments, we recommend integrating elements into the design that meet the supervisory needs of parents, such as adding green spaces and providing areas for waiting and accompaniment, rather than designing play spaces solely from an adult perspective. This approach not only meets the expectations of parents but more importantly, it provides an environment that promotes family interaction and supports children’s autonomous play. Through these adjustments, we hope to better balance reliable and freedom, the perspectives of adults and the needs of children, ultimately fostering the healthy, reliable, and free growth of children in urban environments.

### 6.3 Constructing informal game spaces in child-friendly blocks

China’s high-density urban blocks have large populations with complex demands. Blocks rarely have centrally designed formal game spaces, but more often share comprehensive activity spaces used by people of all ages. Even in child-friendly blocks, the user base for block spaces is diverse, and public spaces on urban blocks are in short supply, making it impractical to restrict a specific group from using a particular public space. When constructing child-friendly blocks in high-density cities, blocks should allocate a certain amount of public space to provide children with reliable formal game spaces. However, in most cases, it is essential to create reliable informal game spaces for various age groups to share. According to children’s personalized game space preferences, providing a variety of styles and elements in informal game spaces is crucial. Such spaces, seemingly not specifically designed for children but still capable of accommodating children’s games and activities, can better meet the leisure needs of various groups, increasing the utilization, vitality, and attractiveness of blocks.

## 7 Discussion and conclusion

Through an in-depth analysis of the empirical study on Baihua Second Road, this research has contributed to revealing the multidimensional characteristics of children’s play preferences in block spaces. The methodology that integrates subjective and objective data not only highlights the complexity of children’s play space choices from different perspectives but also provides targeted guidance for urban planning and design.

1. The study found that children show a significant preference for soft facilities and visually attractive spaces, which is crucial for designing children’s play areas. In high-density urban environments, paying attention to children’s significant preferences for soft facilities and visually attractive spaces during the design process is key to enhancing the children’s play experience. This preference not only reflects children’s basic needs for a reliable and comfortable play environment but also their natural sensitivity to aesthetics and sensory experiences. Soft facilities, such as sponge floors, soft lawns, and moldable sand areas, provide children with a reliable environment that reduces the risk of injury while also encouraging more free and creative play (Drake, 2003). Such facilities meet the needs of children’s physical development and satisfy their desire to explore. At the same time, visually attractive spaces stimulate children’s curiosity and imagination through the use of vibrant colors, unique shapes, and diverse textures, enhancing their motivation to play (Althouse et al., 2003; Korn-Bursztyn, 2012). Colorful play equipment, landscape features of various forms, and vivid ground patterns can not only attract children’s attention but also encourage them to explore different parts of the play space through visual guidance, promoting their sensory development and cognitive learning. Furthermore, incorporating natural elements such as water features and vegetation not only beautifies the play space and increases its visual appeal but also provides children with opportunities to interact with nature. Activities like touching the water surface and observing plant growth allow children to experience nature directly, thereby promoting their awareness and respect for the environment and cultivating a sense of environmental protection.
2. This study further indicates that children have a clear preference for the design of play facilities, emphasizing the importance of integrating and balancing different design elements during the design process. This preference stems not only from their natural attraction to novelty and fun but also reflects their need to explore and their imagination’s natural expression. Therefore, designers planning and designing play spaces should delve into children’s intrinsic needs, creating play facilities that can stimulate children’s curiosity and desire to explore. Designers should use children’s rich imaginations to create appealing play facilities through diverse design shapes (Wilson, 2007). For example, play facilities designed in the shapes of animals and plants from nature can not only attract children’s attention but also spark their interest and curiosity about the natural world. Additionally, adopting sci-fi or fairy-tale themes in design can trigger children’s imagination, allowing them to weave their own stories in play, enhancing the interactivity and educational value of the game. Moreover, designers should also consider the multifunctionality and interactivity of play facilities, providing children with various ways to play and experiences (Sargeant, 2015). For instance, designing transformable or multifunctional play facilities that not only offer basic functions like climbing and sliding but can also be reshaped or functionally changed based on children’s creativity can meet the needs of children of different ages, encouraging cooperation and social interaction among them.
3. The differences between subjective and objective results in the study reveal the complexity of children’s play space choices. These differences not only reflect the diversity of children’s individual preferences under free choice and parental guidance but also highlight the need for urban planning and design practices to more meticulously consider children’s real needs and parental concerns. Children, as independent individuals, have their play space choices influenced by a variety of factors, including but not limited to personal interests, emotional connections, and specific experiences related to the space. For example, children might prefer a play space due to the color or shape of a play facility or because of happy memories shared with family and friends in a particular space (Elkind, 2007; Steger et al., 2021). From parents’ perspective, when influencing children’s choice of play spaces, they often focus more on educational value, and the healthiness of the space. Parents might prefer spaces they believe are more beneficial for children’s growth, such as those that offer physical exercise, environmental education, or social interaction opportunities. These adult-centric choices might not always align with children’s natural inclinations, leading to discrepancies in play space choices. The existence of these differences requires urban planners and designers to not only provide educational, and healthy play spaces when conceiving child-friendly neighborhoods but also to consider how to spark children’s interests and desires to explore. Creating play spaces that meet parents’ expectations and attract children’s active participation is key to achieving a child-friendly urban environment (Agarwal et al., 2021). This may mean introducing more participatory design processes in planning and design, allowing both children and parents to be involved in the creation of play spaces, together discussing and deciding on the space’s functions, shapes, and design elements.
4. This study underscores the importance of creating informal play spaces within child-friendly neighborhoods. These spaces not only provide children with opportunities for free play and exploration but also enhance the vibrancy and attractiveness of block spaces, fostering interactions and connections among people of different ages within the community. The design philosophy behind informal play spaces aims to offer an open environment that meets children’s play needs without being limited to specific play facilities, thus encouraging children to independently explore and invent ways to play. Informal play spaces support various types of children’s activities through flexible and varied designs, including but not limited to traditional physical activities, role-playing games, nature exploration, and artistic creation. For instance, open areas can serve as places for running and jumping, while a corner might be designated for nature exploration, such as a small botanical garden or a micro-ecosystem pond, allowing children to directly interact with soil, water, plants, and small animals, and experience the mysteries of nature. Additionally, movable play elements like sandboxes, blocks, and drawing boards can provide materials for children to unleash their creativity and can be reconfigured and arranged as needed for activities (Solomon, 2014). The presence of such spaces helps develop children’s ability to think independently and solve problems because they provide a play activities environment free from strict adult rules, allowing children to set their own rules and negotiate interaction modes with peers. This openness and flexibility not only promote the development of children’s social skills and team spirit but also enhance their perception and adaptability to their surroundings. Moreover, the design of informal play spaces must consider accessibility to ensure all children, including those with different abilities, can benefit from them (Casey, 2017). For example, ensuring that the ground is level, materials are harmless, corners are rounded, and providing sufficient visibility for parents or guardians to conveniently supervise children’s play.

By comprehensively understanding the complexity of children’s choices of play spaces in urban block environments, this research makes a significant contribution to the field of environmental psychology. It not only reveals how spatial elements affect children’s behaviors and preferences but also emphasizes the subjectivity of children as users of urban spaces and their active roles in urban environments. This perspective helps to advance more child-centered urban planning and design research, promoting the creation of more inclusive and participatory urban spaces. For instance, by optimizing urban space design and adding attractive children’s play areas, children’s activity frequency and satisfaction in urban environments can be effectively increased, thereby fostering children’s holistic development. Additionally, this study highlights the importance of community involvement, encouraging parents, schools, and community groups to participate in the planning and construction of child-friendly spaces to ensure these areas truly meet children’s needs and expectations.

### 7.1 Research limitations

Despite providing valuable insights into children’s play space preferences and their relationship with urban spatial elements, this study has some limitations. First, the research objectives is limited to a single block in Shenzhen, which may not fully represent the play preferences of children from other cultural and geographical backgrounds. Second, the study relies on observations of specific spaces and facilities and questionnaire surveys, which could be influenced by participants’ subjective willingness and interpretation biases. Lastly, there is room for improvement in the research methodology. In this study, we primarily used a negative binomial regression model, judging the robustness and credibility of the regression results by observing the size of the *p*-value. We did not employ triangulation for further argumentation, which is a shortfall of this study. In future research, we will use this method for deeper exploration. Therefore, future studies should consider cross-regional, multicultural comparative research, and the adoption of more diversified data collection and analysis methods to further enrich the understanding of children’s play space preferences.

## Data availability statement

The original contributions presented in the study are included in the article/supplementary material, further inquiries can be directed to the corresponding author.

## Ethics statement

The studies involving humans were approved by Ethics Committee of Beijing Forestry University. The studies were conducted in accordance with the local legislation and institutional requirements. Written informed consent for participation in this study was provided by the participants’ legal guardians/next of kin.

## Author contributions

YW: Conceptualization, Data curation, Methodology, Software, Visualization, Writing – original draft. YNS: Conceptualization, Data curation, Funding acquisition, Methodology, Project administration, Resources, Writing – original draft, Writing – review & editing. YHS: Data curation, Funding acquisition, Investigation, Methodology, Software, Visualization, Writing – original draft, Writing – review & editing. TH: Data curation, Methodology, Resources, Software, Supervision, Visualization, Writing – original draft.
